# Renal Failure Caused by Malakoplakia Lesions of the Urinary Bladder

**DOI:** 10.5812/numonthly.18522

**Published:** 2014-07-05

**Authors:** Konstantinos Stamatiou, Eleni Chelioti, Aikaterini Tsavari, Kalliroi Koulia, Alexia Papalexandrou, Evdokia Efthymiou, Maria Tsilivigkou, Thivi Vasilakaki

**Affiliations:** 1 Department of Urology, “Tzaneion” General Hospital of Piraeus, Athens, Greece; 2 Department of Nephrology, “Tzaneion” General Hospital of Piraeus, Athens, Greece; 3 Department of Pathology, “Tzaneion” General Hospital of Piraeus, Athens, Greece

**Keywords:** Malakoplakia, Inflammatory Disease, Urinary Bladder, Kidney Failure

## Abstract

Μalakoplakia is a rare inflammatory condition of the urogenital tract. The most frequently affected organ is urinary bladder. This condition has features of a granulomatous inflammation, the pathogenesis of which is not well understood. In this study, we presented a case of urinary bladder malakoplakia associated with advanced obstructive uropathy and renal failure.

## 1. Introduction

Malakoplakia is a respectively rare condition which was first described in 1902 by Michaelis and Gutmann. It affects both sexes (especially women) and mostly people over 40 years old. It rarely occurs in the urogenital tract. When bladder is affected, symptoms are not specific and they mimic those of cystitis (1). Both etiology and pathogenesis have remained unknown; however, it is presumed that development of malakoplakia is associated with macrophages malfunction. More precisely, defects in phagocytic or degradative functions of histiocytes in response to gram-negative coliforms (*E. coli* or *Proteus*) results in a chronic inflammatory state which manifests itself as plaque or papule formation (2). The lesions are characterized by presence of large macrophages; foamy histiocytes (known as cells von Hansemann) containing Michaelis-Gutmann bodies. The last event is intracellular deposition of iron and calcium, produced by accumulation of partially digested bacteria in macrophages. Macroscopically, as clinically, malakoplakia can simulate tumors or abscesses; however, the typical cystoscopy appearance of the affected bladder is characterized by yellow soft tiny plates. This entity is associated with a history of immunosuppression due to lymphoma, diabetes mellitus, renal transplantation, or it can occur due to long-term therapy with systemic corticosteroids or more frequently in patients with a prior infection of *E. coli* (2).

## 2. Case Presentation

**Figure 1.**
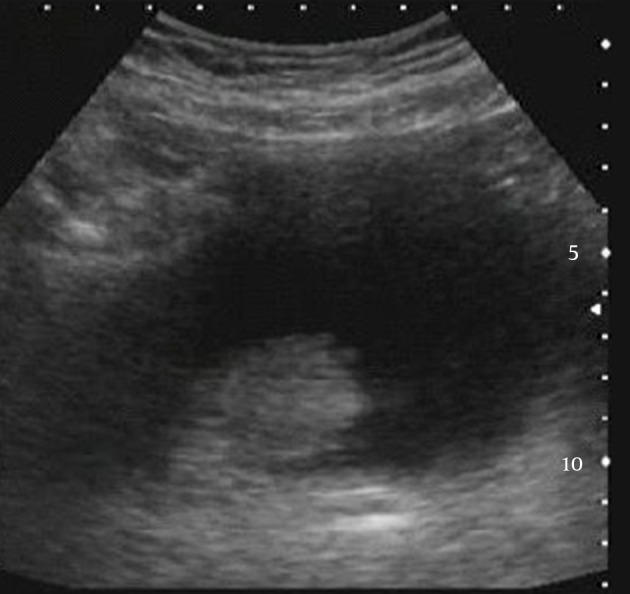
Transabdominal Ultrasound: Abnormal Thickening of Luminal Bladder, With Presence of Nodules.

A 72-year-old Caucasian man was admitted to the emergency department with reported inability to urinate and fever. He also reported weakness, exhaustion, and irritative voiding symptoms for approximately one month. His current medical history included diabetes, vascular disease, and recurrent urinary tract infections (UTI). In addition, he underwent bladder biopsy due to abnormal formation of bladder mucosa (diagnosed as nonspecific inflammation) and transurethral prostatectomy, three years prior to the admission. Besides hypotension, other vital signs were normal. Upon investigation, he was diagnosed with obstructive uropathy with bilateral hydronephrosis, concomitant renal insufficiency, (serum creatinine: 21 mg/dL) and anemia (hematocrit: 19%). The serum glucose level was 300 mg/dL and there was no leukocytosis. Urinalysis revealed hematuria, pyuria, and bacteriuria. Ultrasound scan of the abdomen showed abnormal thickening of the bladder wall with presence of nodules and small functional capacity, while no residual urine was found (Figure 1). On clinical examination, the prostate gland was smooth and small. The patient underwent dialysis sessions (due to the underlying hyperkalemia and anuria) and nephrostomy tubes were placed in both sides. After stabilization of renal function the patient underwent cystoscopy, which revealed atypical polypoid mucosal lesions of the bladder. They resembled neoplastic masses and scattered red-yellow nodules of 2-3 and 5 cm, located mainly in the trigonal area, left ureteric orifice, posterior wall, and bladder roof.

**Figure 2.**
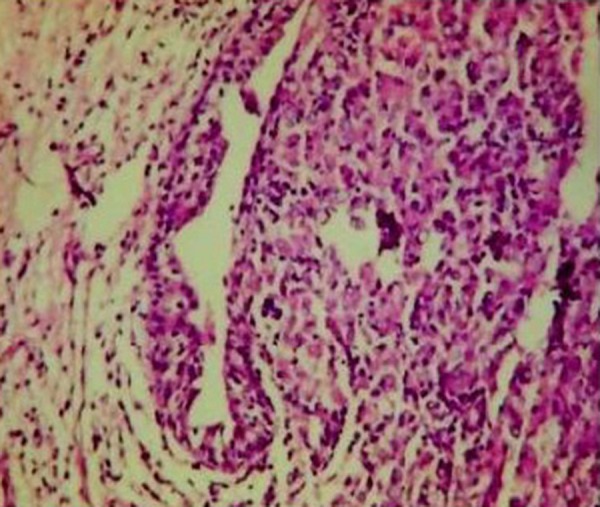
Pathognomonic Histological Inflammatory Infiltrates (ΗΕx100)

**Figure 3.**
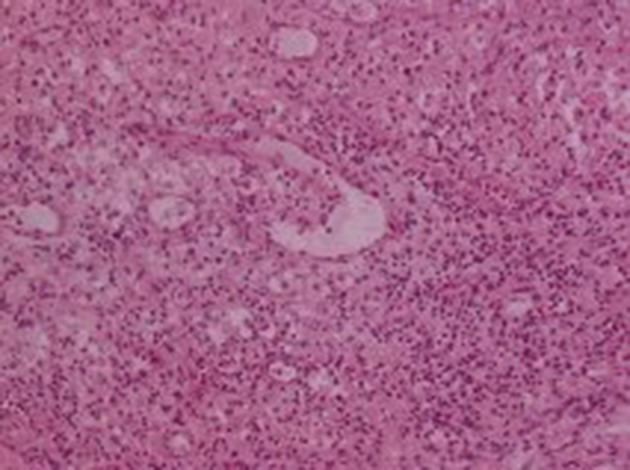
Malakoplakia of the Urinary Bladder: Aggregates of Large Macrophages (HEx200)

**Figure 4.**
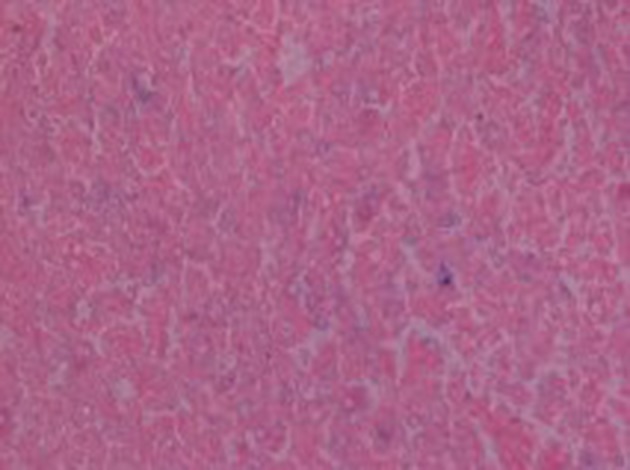
Michaelis-Gutmann Bodies, Perl Stain Positive (x400)

He subsequently underwent transurethral resection of the bladder lesions. Cystectomy was not performed, because the patient was too frail to undergo such a long surgery. On the histologic examination of the bladder specimens, aggregates of large macrophages with fine eosinophilic granular cytoplasm (von Hansemann cells) admixed with basophilic inclusions (Michaelis-Gutmann bodies) and infiltrated by dense collections of lymphocytes, as well as plasma cells, were seen in lamina propria of urinary bladder (Figures 2, 3 and 4). Macrophages were immunohistochemically negative for cytokeratin and positive for CD68. Immunohistochemical examination of proliferative activity, measured by Ki-67 in malakoplakia, was negative. Despite the absence of bacteria in the urinalysis, the patient received quinolones for three months, according to the current evidence. However, the patient remained with UTIs, chronic hydronephrosis, renal failure, and permanent bladder catheter. Finally, he died eight months later, after a further worsening of renal failure and complications of the cardiovascular system.

## 3. Discussion

Malakoplakia is a benign self-limiting condition with usually good prognosis (3). Initial assessment includes UTI treatment and surgical removal of the lesion. Very few cases of renal failure associated with multifocal malakoplakia have been described in the literature (3). Since symptoms are typically unpleasant and not self-limiting, diagnosis can be made in early stages in most of the patients (4). Reasons of delayed diagnosis in the present case were practically unknown. Given the chronicity of symptoms, it can be assumed that malakoplakia had been present at the time of first bladder biopsy. Failure in diagnosis might be due to insufficient biopsy materials and concomitant absence of pathognomonic findings (such as microscopic acidophilic foamy histiocytes and Michaelis-Gutmann bodies). However, when clinical suspicion is strong (despite absence of typical pathognomonic findings), CD68-positive immunohistochemical staining confirms the diagnosis (5). Although this inflammatory condition is chronic and spreads slowly through the bladder mucosa, it may progress rapidly in the presence of underlying illnesses and conditions such as autoimmune diseases or immunodeficiency (5).

In conclusion, bladder malakoplakia should be carefully considered in patients presenting recurrent UTIs, who have not responded to the treatment and have been suspicious in cystoscopic findings. An early diagnosis and early treatment with antibiotics may be useful for preventing the development of possible complications. Patients similar to the present case should be closely monitored.
